# Mycetoma Leading to Effusive Constrictive Pericarditis in an Immunocompetent Individual: A Rare Encounter

**DOI:** 10.7759/cureus.73287

**Published:** 2024-11-08

**Authors:** Juan C Rivera-Martinez, Philip S Owen, Sami Baddoura, Mohammed Hassan

**Affiliations:** 1 Internal Medicine, Lakeland Regional Health Medical Center, Lakeland, USA; 2 Cardiology, Lakeland Regional Health Medical Center, Lakeland, USA; 3 Cardiothoracic Surgery, Lakeland Regional Health Medical Center, Lakeland, USA

**Keywords:** constrictive pericarditis, diagnostic evaluation, nocardial pericarditis, pericardial effusion, pericardiectomy

## Abstract

Nocardial infections are rare but serious, often leading to systemic and cardiopulmonary complications. This is the first reported case of *Nocardia beijingensis* causing constrictive pericarditis in an immunocompetent individual. We present a 37-year-old Caucasian female patient with no significant medical history who developed pericarditis symptoms after handling crates from China. Despite initial treatment for presumed viral pericarditis, her condition worsened. Further investigation identified *Nocardia beijingensis* as the cause of effusive constrictive pericarditis. The patient underwent a pericardiectomy, which resolved her symptoms. This case demonstrates the need to consider rare pathogens in cases of refractory pericarditis, regardless of the patient's immune status. Clinicians should maintain a high level of suspicion for uncommon infections in progressively worsening cases and adopt a multidisciplinary diagnostic approach for timely intervention.

## Introduction

Pericarditis can be attributed to inflammatory conditions, immune-mediated factors [1,2], and infectious agents, including viruses, bacteria, fungi, and parasites. Constrictive pericarditis, a rare complication, is most commonly associated with tuberculous [3] or viral infections. The diagnosis involves a combination of clinical symptoms, cardiac auscultation, and electrocardiographic and echocardiographic findings [4-7]. Pericardial disease secondary to *Nocardia beijingensis* has been documented in immunocompromised patients [8]; however, to our knowledge, no cases have been reported in immunocompetent individuals. This case details the challenging diagnostic process and emphasizes the need to consider rare pathogens even in immunocompetent patients.

## Case presentation

**Figure 1:**
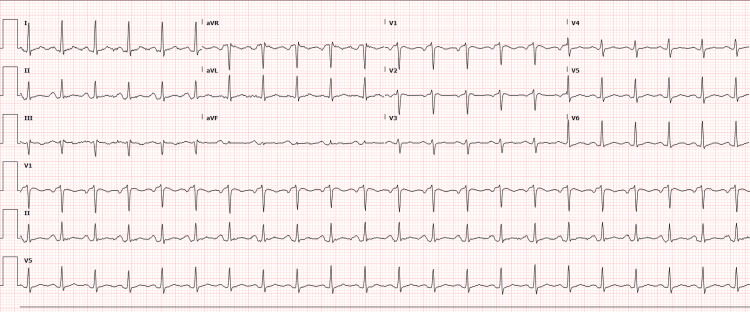
Electrocardiogram (EKG) on initial presentation The EKG shows sinus tachycardia with a regular rhythm and a heart rate of 130 bpm. P-waves precede each QRS complex, indicating sinus origin, with narrow QRS complexes and normal PR intervals. bpm: beats per minute.

A 37-year-old Caucasian female patient, with no significant past medical history, presented to the emergency department with a one-month history of pleuritic substernal chest pain radiating to the upper back and dyspnea on mild activities. She also reported intermittent fevers and night sweats during this period. She works in a hot warehouse with a large fan for ventilation, opening crates and assembling parts from China. On physical examination, no significant murmurs or rubs were appreciated on auscultation. The electrocardiogram showed sinus tachycardia at 130 beats per minute (bpm) (Figure 1). The initial echocardiography demonstrated a left ventricular ejection fraction of 55-60% and a small-to-moderate pericardial effusion. The initial laboratory analysis revealed leukocytosis with significant neutrophil shift, elevated inflammatory markers, and thrombocytosis. The patient was managed with ibuprofen and colchicine with a presumptive diagnosis of viral pericarditis and was discharged with instructions for outpatient follow-up.

**Figure 2:**
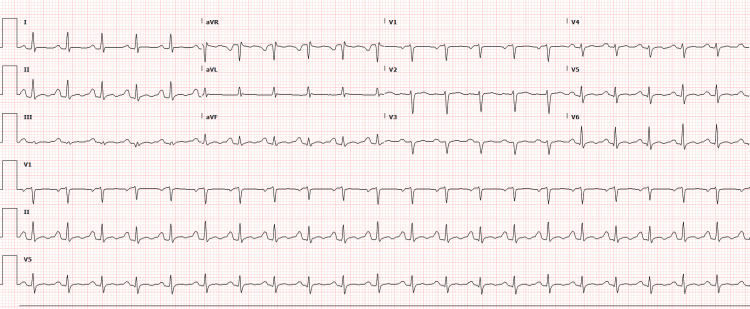
Electrocardiogram (EKG) 12 days post-discharge The EKG shows sinus tachycardia with a regular rhythm and a heart rate of 128 bpm. P-waves precede each QRS complex, indicating sinus origin, with narrow QRS complexes and normal PR intervals. bpm: beats per minute.

Twelve days post-discharge, the patient was reevaluated in the outpatient cardiology clinic and subsequently readmitted with tachycardia, worsening pleuritic chest pain radiating to the shoulders, progressive dyspnea, fatigue, and tiredness. On physical examination, she was noted to be pale, ill-appearing, and tachycardic. Auscultation was unremarkable. An electrocardiogram once again revealed sinus tachycardia at 128 bpm (Figure 2). White blood cells remained elevated with a left shift. Follow-up echocardiograms revealed a moderate-to-large pericardial effusion with early tamponade. Persistent symptoms necessitated a pericardial window procedure with biopsy and drain placement, resulting in clinical improvement. Three hundred fifty milliliters of murky pericardial fluid was retrieved. The cytology indicated severe acute inflammation without malignancy. Cultures ultimately identified *Nocardia beijingensis*. The patient was treated with meropenem and trimethoprim-sulfamethoxazole. 

Extensive testing for autoimmune diseases, connective tissue disorders, blood cultures, and viral, bacterial, and fungal pathogens was unremarkable. CD4 count was recorded at 1,798 cells/mcL. The patient was discharged with instructions for outpatient follow-up.

**Figure 3:**
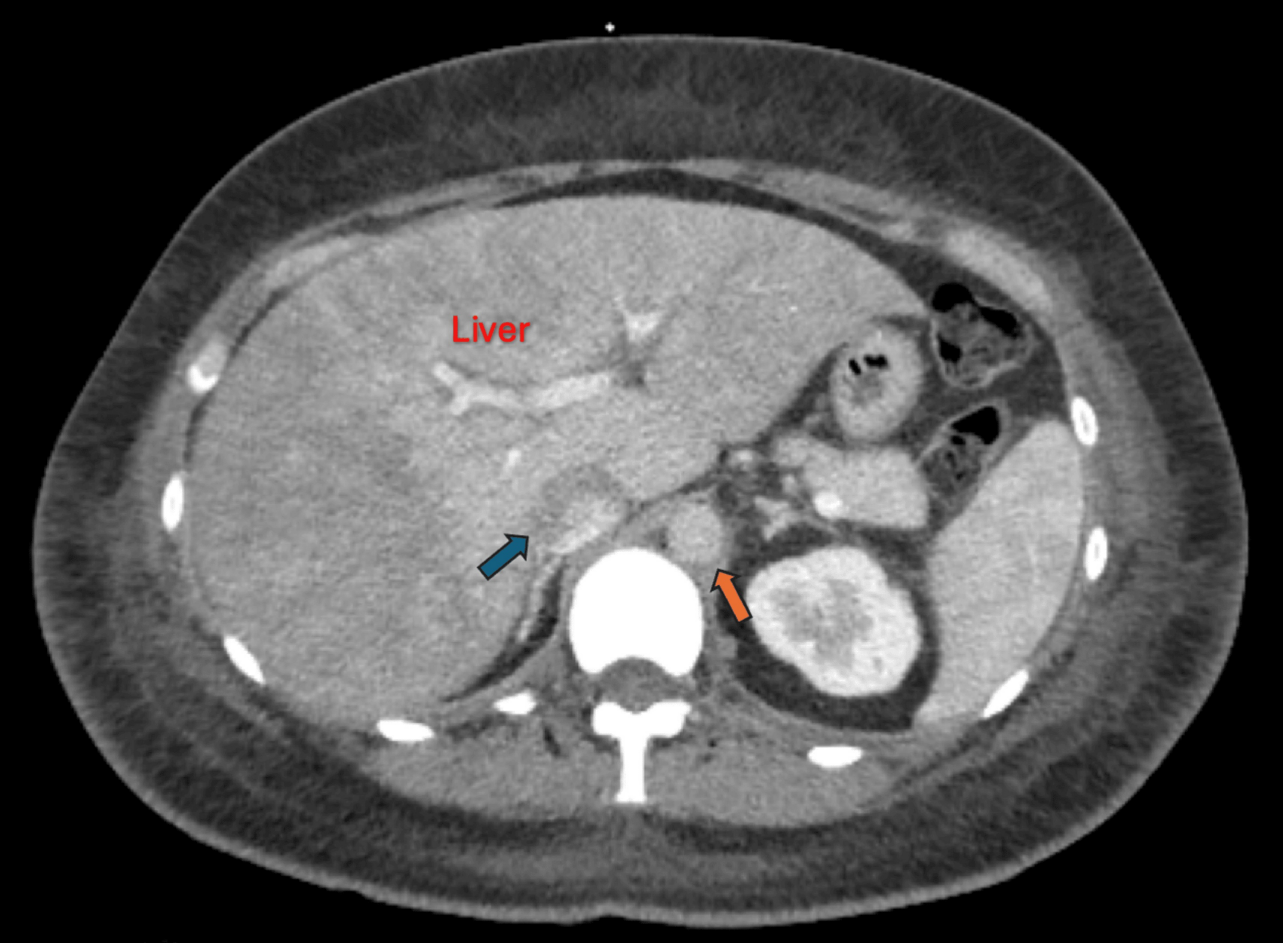
Computerized tomography (CT) of the abdomen with contrast (axial cut) at the level of the upper abdomen The image shows congestive hepatomegaly, with the blue arrow pointing to the distended inferior vena cava. Together, this indicates elevated right heart pressures. For reference, the orange arrow marks the aorta.

**Figure 4:**
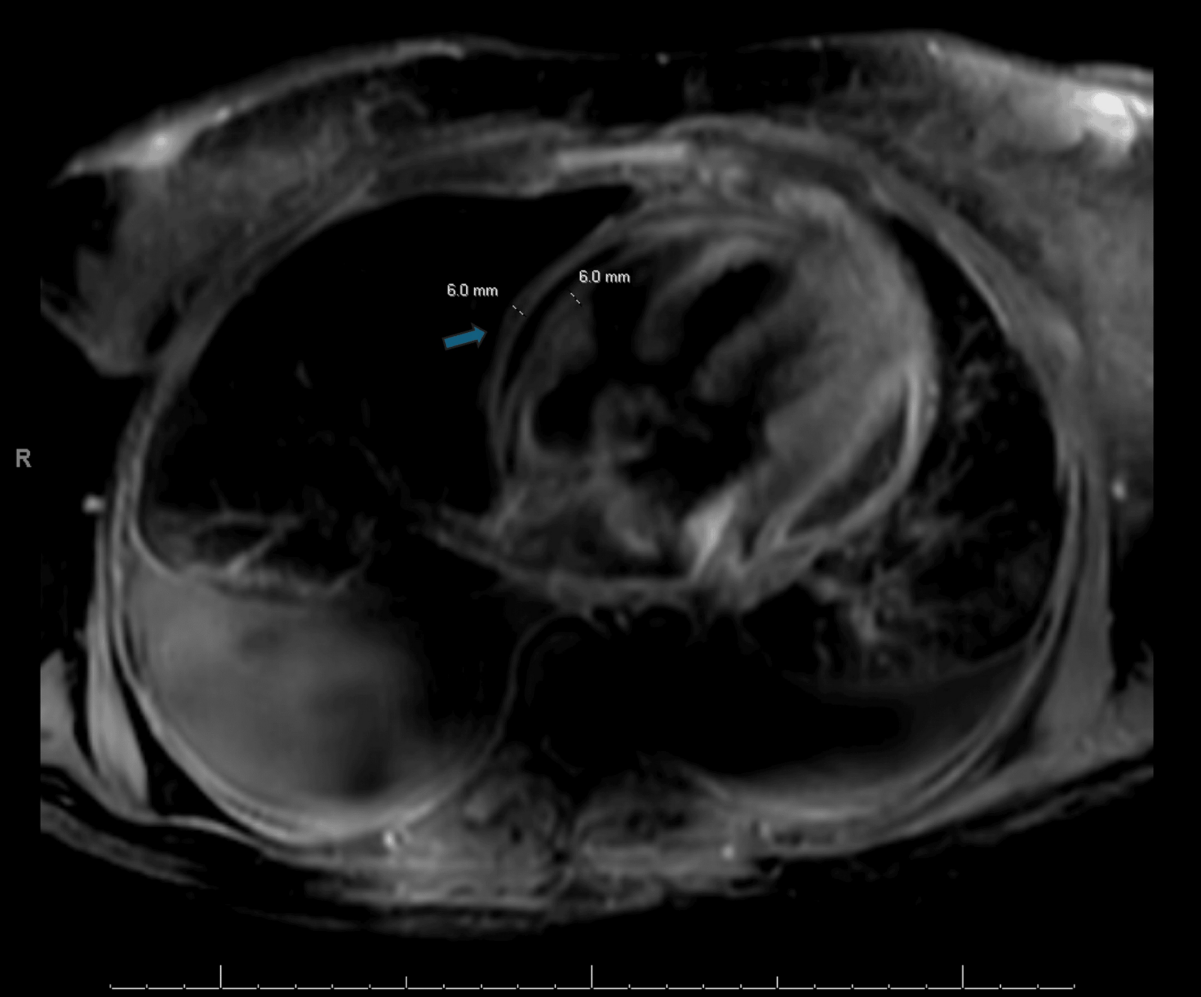
Cardiac magnetic resonance imaging (MRI) of the heart (four-chamber view) The image demonstrates pericardial thickening, indicated by the blue arrow. In constrictive pericarditis, pericardial thickening reflects inflammation and fibrosis, resulting in a rigid and inelastic pericardium. This rigidity restricts diastolic filling, limiting the heart’s capacity to expand during relaxation. Consequently, filling pressures rise, which aligns with the clinical presentation of constrictive pericarditis.

**Figure 5:**
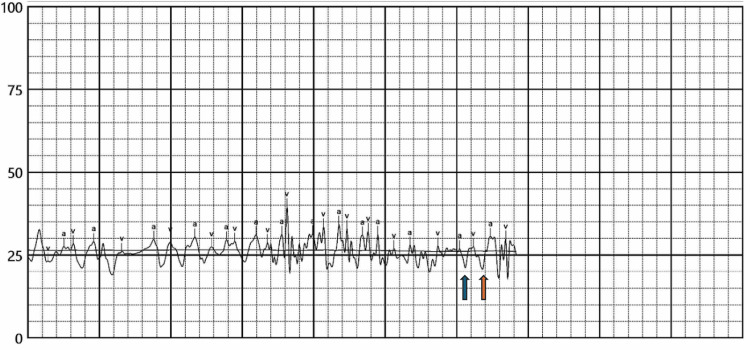
Right atrial pressure waveform (right heart catheterization) The blue arrow points at the prominent "x" descent. The orange arrow points at the prominent "y" descent. These findings are consistent with constrictive pericarditis.

**Figure 6:**
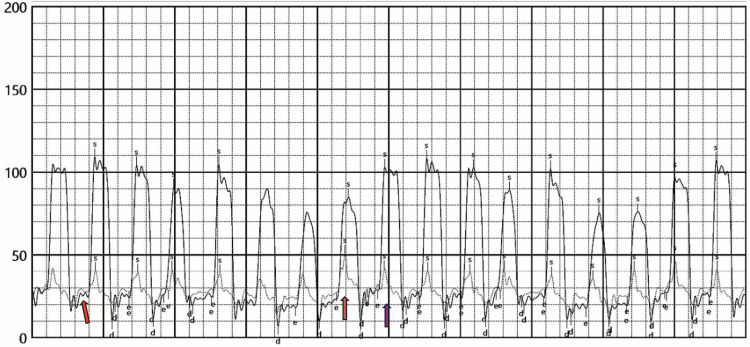
Simultaneous left and right ventricular pressure recordings (right and left cardiac catheterization) The complete line represents left ventricular (LV) pressure, while the dotted line shows right ventricular (RV) pressure tracings. In constrictive pericarditis, during inspiration (orange arrow), the RV enlarges while the LV decreases in volume. During expiration (purple arrow), the RV decreases in size as the LV expands. This reciprocal change, known as RV-LV respiratory systolic discordance, reflects impaired ventricular interaction caused by the rigid pericardium. The red arrow points to the equalization of end-diastolic pressures in both ventricles. Together, these findings (RV-LV respiratory systolic discordance and equalization of end-diastolic pressures) are hallmark features of constrictive pericarditis.

**Figure 7:**
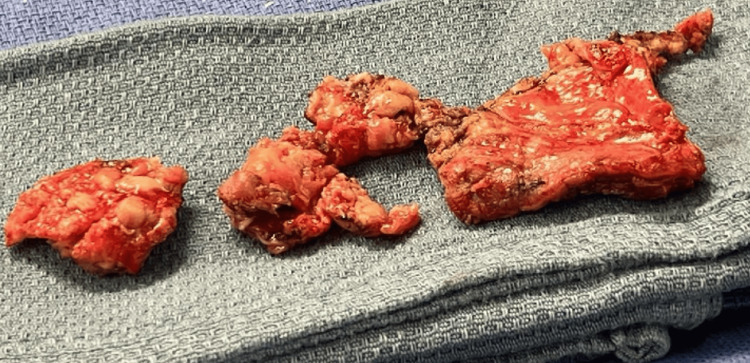
Pathology samples post-pericardiectomy The image shows a thickened and fibrotic pericardium, consistent with a mycetoma.

**Figure 8:**
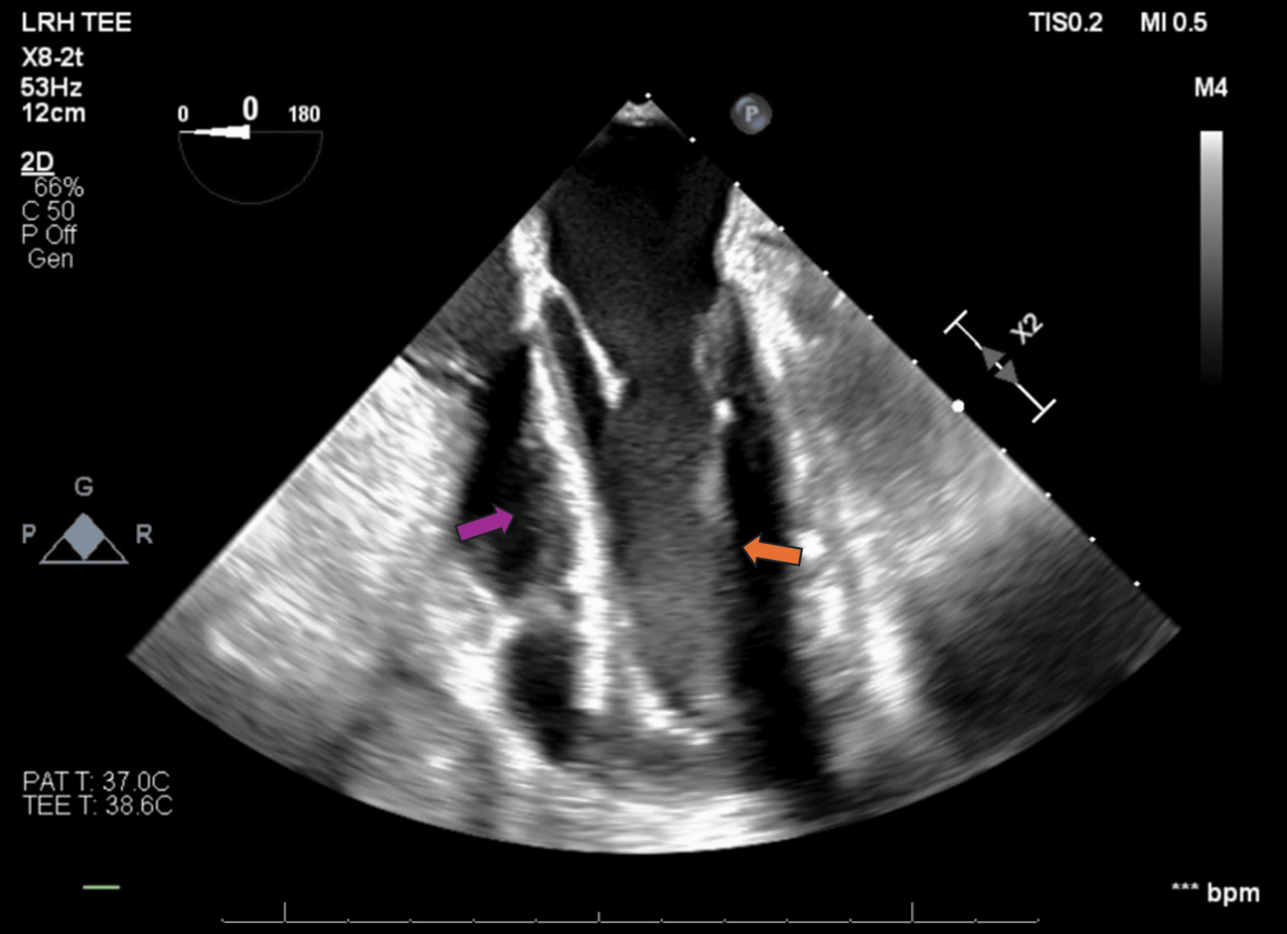
Transesophageal echocardiogram pre-pericardiectomy (mid-esophageal four-chamber view) The image reveals compression of both the right and left ventricles. The purple arrow indicates the compression of the right ventricle, while the orange arrow indicates the compression of the left ventricle.

**Figure 9:**
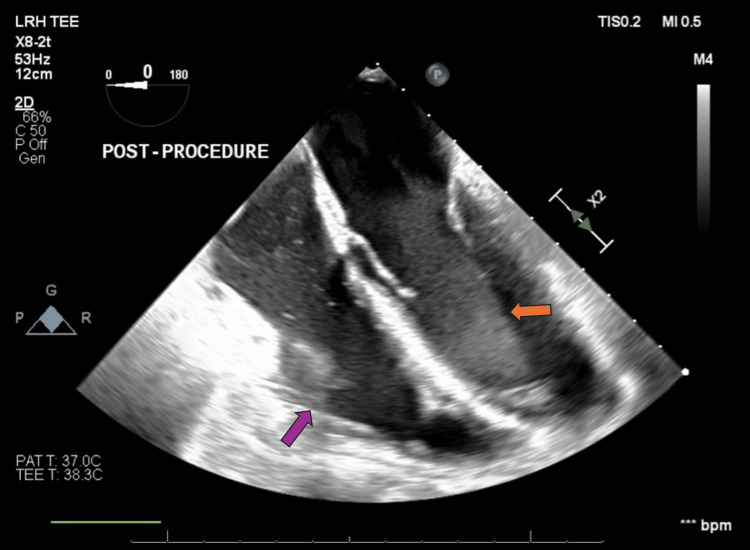
Transesophageal echocardiogram post-pericardiectomy (mid-esophageal four-chamber view) The purple arrow points to the right ventricle, now exhibiting normal size and filling, indicating the resolution of the previous compression. The orange arrow points at the left ventricle, also showing improved contour, suggesting restored ventricular dynamics post-procedure. These findings suggest successful alleviation of constrictive physiology.

Three days post-discharge, the patient returned to the emergency department for a third time with worsening dyspnea. The physical examination revealed elevated jugular venous pressure with Kussmaul's sign, significant bilateral lower extremity edema, and abdominal distention. Computed tomography (CT) of the abdomen with contrast demonstrated congestive hepatomegaly and a distended inferior vena cava (Figure 3), indicative of elevated right heart pressures. Repeat echocardiography showed persistent pericardial effusion despite the prior pericardial window procedure (Video 1). Cardiac magnetic resonance imaging (MRI) revealed a pericardial thickness of 6 mm (Figure 4). Right and left heart catheterization showed no coronary artery disease, diastolic pressure equalization (approximately 25 mmHg), elevated right atrial pressure with prominent "x" and "y" descends (Figure 5), dissociation of intracardiac and intrathoracic pressures, and discordance of left ventricular and right ventricular systolic area indices (Figure 6), consistent with constrictive pericarditis. The patient subsequently underwent anterior and diaphragmatic pericardiectomy, and the histopathological examination confirmed extensive acute and chronic inflammation and a fibrinous pericardium consistent with mycetoma (Figure 7). Pre-procedure (Figure 8, Video 2) and post-procedure (Figure 9, Video 3) transesophageal echocardiograms were obtained, the latter revealing significant right ventricular expansion. 

**Video 1:** Transthoracic echocardiogram (apical four-chamber view) This transthoracic echocardiogram shows fluid accumulation in the pericardial space despite a previous pericardial window procedure.

**Video 2:** Transesophageal echocardiogram pre-pericardiectomy (mid-esophageal four-chamber view) The video shows compression of both the right and left ventricles.

**Video 3:** Transesophageal echocardiogram post-pericardiectomy (mid-esophageal four-chamber view) The video shows improved cardiac chamber fillings, indicating the resolution of the previous compression.

## Discussion

Nocardial pericarditis is a rare but life-threatening condition. *Nocardia *species are aerobic actinomycetes that typically cause systemic infections in immunocompromised individuals [9]. However, this patient had no history of autoimmune disease or immune system compromise, making the diagnosis more challenging and underscoring the need for a high index of suspicion in atypical presentations. Nocardia species are fastidious, known for their slow growth [10], and can be easily overlooked if not specifically sought in culture.

The diagnosis of pericarditis generally involves ruling out common causes such as viral infections and autoimmune conditions and is primarily based on clinical assessments. In this case, the patient's lack of response to standard anti-inflammatory treatment and the rapid progression to pericardial tamponade necessitated a comprehensive evaluation for less common etiologies, ultimately leading to a pericardial window. The identification of *Nocardia beijingensis* in the pericardial fluid was unexpected but crucial for guiding appropriate therapeutic management.

She developed rapidly progressive effusive constrictive pericarditis as evidenced by an echocardiogram and right and left heart catheterization. The effusive constrictive pericarditis was secondary to the nocardial infection, which caused persistent inflammation and fibrosis with mycetoma of the pericardium. The timely identification of *Nocardia beijingensis* in the pericardial fluid allowed for targeted antimicrobial therapy, initially improving the patient’s condition. However, the delayed presentation, diagnosis, and treatment likely contributed to the progression of constrictive pericarditis.

## Conclusions

This case emphasizes the need for a thorough evaluation of persistent pericarditis, including pericardiocentesis and fluid analysis. Although rare, *Nocardia beijingensis* pericarditis should be considered, especially in patients who do not respond to standard treatments. Assessing immune competence and screening for autoimmune conditions are also important. Prompt recognition and initiation of antimicrobial therapy are vital. A multidisciplinary approach involving cardiologists, infectious disease specialists, and microbiologists is key for accurate diagnosis and effective treatment.
